# ZNF677 suppresses renal cell carcinoma progression through N6‐methyladenosine and transcriptional repression of CDKN3

**DOI:** 10.1002/ctm2.906

**Published:** 2022-06-09

**Authors:** Aolin Li, Congcong Cao, Ying Gan, Xiaofei Wang, Tianyu Wu, Quan Zhang, Yuchen Liu, Lin Yao, Qian Zhang

**Affiliations:** ^1^ Department of Urology Peking University First Hospital Beijing China; ^2^ Institute of Urology Peking University Beijing China; ^3^ National Urological Cancer Center Beijing China; ^4^ Beijing Key Laboratory of Urogenital Diseases (male) Molecular Diagnosis and Treatment Center Beijing China; ^5^ Guangdong Key Laboratory of Systems Biology and Synthetic Biology for Urogenital Tumors Shenzhen Second People's Hospital The First Affiliated Hospital of Shenzhen University Shenzhen China

**Keywords:** CDKN3, dCas13b, m^6^A, RCC, ZNF677

## Abstract

**Background:**

Studies on biological functions of N6‐methyladenosine (m^6^A) modification in mRNA have sprung up in recent years. Previous studies have reported m^6^A can determine mRNA fate and play a pivotal role in tumour development and progression. The zinc finger protein 677 (ZNF677) belongs to the zinc finger protein family and possesses transcription factor activity by binding sequence‐specific DNA.

**Methods:**

The expression of ZNF677 and its clinicopathological impact were evaluated in renal cell carcinoma (RCC) patients. The m^6^A level of ZNF677 was determined by m^6^A methylated RNA immunoprecipitation‐sequencing (MeRIP‐seq) and MeRIP‐qPCR in RCC tissues and adjacent normal tissues. RNA immunoprecipitation‐qPCR (RIP‐qPCR) and luciferase assays were performed to identify the targeted effect of IGF2BP2 and YTHDF1 on ZNF677. RCC cells and subcutaneous models uncovered the role of ZNF677 methylated by CRISPR/dCas13b‐METTL3 in tumour growth. ZNF677‐binding sites in the CDKN3 promoter were investigated by chromatin immunoprecipitation (ChIP) and luciferase assays.

**Results:**

ZNF677 is frequently downregulated in RCC tissues and its low expression is associated with unfavourable prognosis and decreased m^6^A modification level. Further, we find the m^6^A‐modified coding sequence (CDS) of ZNF677 positively regulates its translation and mRNA stability via binding with YTHDF1 and IGF2BP2, respectively. Targeted specific methylation of ZNF677 m^6^A by CRISPR/dCas13b‐METLL3 system can significantly increase the m^6^A and expression level of ZNF677, and dramatically inhibit cell proliferation and induce cell apoptosis of RCC cells. In addition, ZNF677 exerted its tumour suppressor functions in RCC cells through transcriptional repression of CDKN3 via binding to its promoter. In vitro and clinical data confirm the negative roles of ZNF677/CDKN3 in tumour growth and progression of RCC.

**Conclusion:**

ZNF677 functions as a tumour suppressor and is frequently silenced via m^6^A modification in RCC, which may highlight m^6^A methylation‐based approach for RCC diagnosis and therapy.

## Graphical abstract

1. ZNF677 is frequently silenced via m^6^A modification in RCC.
2. The m^6^A modified CDS of ZNF677 positively regulates its translation and stability via binding with YTHDF1 and IGF2BP2, respectively.
3. ZNF677 exerted its tumor suppressor functions in RCC cells through transcriptional repression of CDKN3 via binding to its promoter.

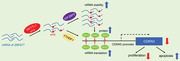

## 1 BACKGROUND

Renal cell carcinoma (RCC) is the most common malignancy of the genitourinary system. Renal cancer is the sixth most common cancer in men, with 65 000 new cases and 15 000 deaths a year, according to the latest cancer data from the United States.^1^ Although the oncology research and surgical treatment of RCC has developed rapidly, the prognosis of RCC has not improved significantly. For local RCC, 20%–30% of patients relapse after initial surgical treatment, and no treatment has been shown to reduce tumour recurrence and improve prognosis.^2^ In recent years, targeted agents have been shown to prolong survival and prognosis in patients with metastases, but the median survival is still less than 3 years.^3^ In addition, drug resistance and economic burden are two major problems in clinical practice. Therefore, the study on the pathological mechanism and new therapeutic targets of RCC is still a challenging exploration.

The modified RNA plays a crucial role in the posttranscriptional regulation of gene expression. In eukaryotes, N6‐methyladenosine (m^6^A) is the most common internal modification, and its abundance has been found to account for 0.1%–0.4% of the total adenosine residue.^4^, ^5^ In general, m^6^A is highly conserved between humans and mice, located in the 3′‐terminal noncoding region, near the stop codon and long internal exons, and associated with altered RNA stability, splicing, intracellular distribution and translation.^4^, ^6^, ^7^ The cellular m^6^A state is mediated by a set of genes called ‘writers’ (WTAP, METTL3 and METTLL4), ‘erasers’ (FTO and ALKBH5) and ‘readers’ (YTHDF1/2/3, IGF2BP2/3, YTHDCL and YTHDC2).^8^, ^9^, ^10^, ^11^, ^12^, ^13^, ^14^ The writer forms a multisubunit methyltransferase complex that upregulates m^6^A levels, while the eraser is m^6^A demethylase, making this event reversible.^4^, ^7^, ^15^, ^16^ In recent years, m^6^A RNA modification on the sixth nitrogen atom of RNA molecule adenine has become one of the hot topics in various human diseases such as hypertension,^17^ cardiac hypertrophy,^18^ viral infection,^19^ diabetes^20^ and cancers.^21^, ^22^ Another research showed that m^6^A modification could promote the translation of PLOD2 protein after YTHDF1 recognition, and then promoting the occurrence and development of RCC.^24^ In addition, one study demonstrated that zinc finger protein 677 (ZNF677) functioned as a tumour suppressor and was frequently silenced via promoter methylation in thyroid cancer.^25^ However, the RNA m^6^A expression patterns and their relevant mechanisms in RCC remain largely unknown.

In the present study, we found that the ZNF677 m^6^A and expression level were significantly decreased in RCC tissues compared to adjacent normal tissues. Based on preliminary experimental results, we demonstrated that the methylation of coding sequence (CDS) in ZNF677 can regulate its mRNA stability and translation via recruitment of different m^6^A reader proteins. Further, ZNF677 can also bind to the promoter of its target CDKN3 to regulate cell proliferation and apoptosis in RCC cells.

## 2 RESULTS

### 2.1 Downregulation of ZNF677 is associated with unfavourable prognosis and decreased m^6^A methylation modification levels in RCC

**FIGURE 1:**
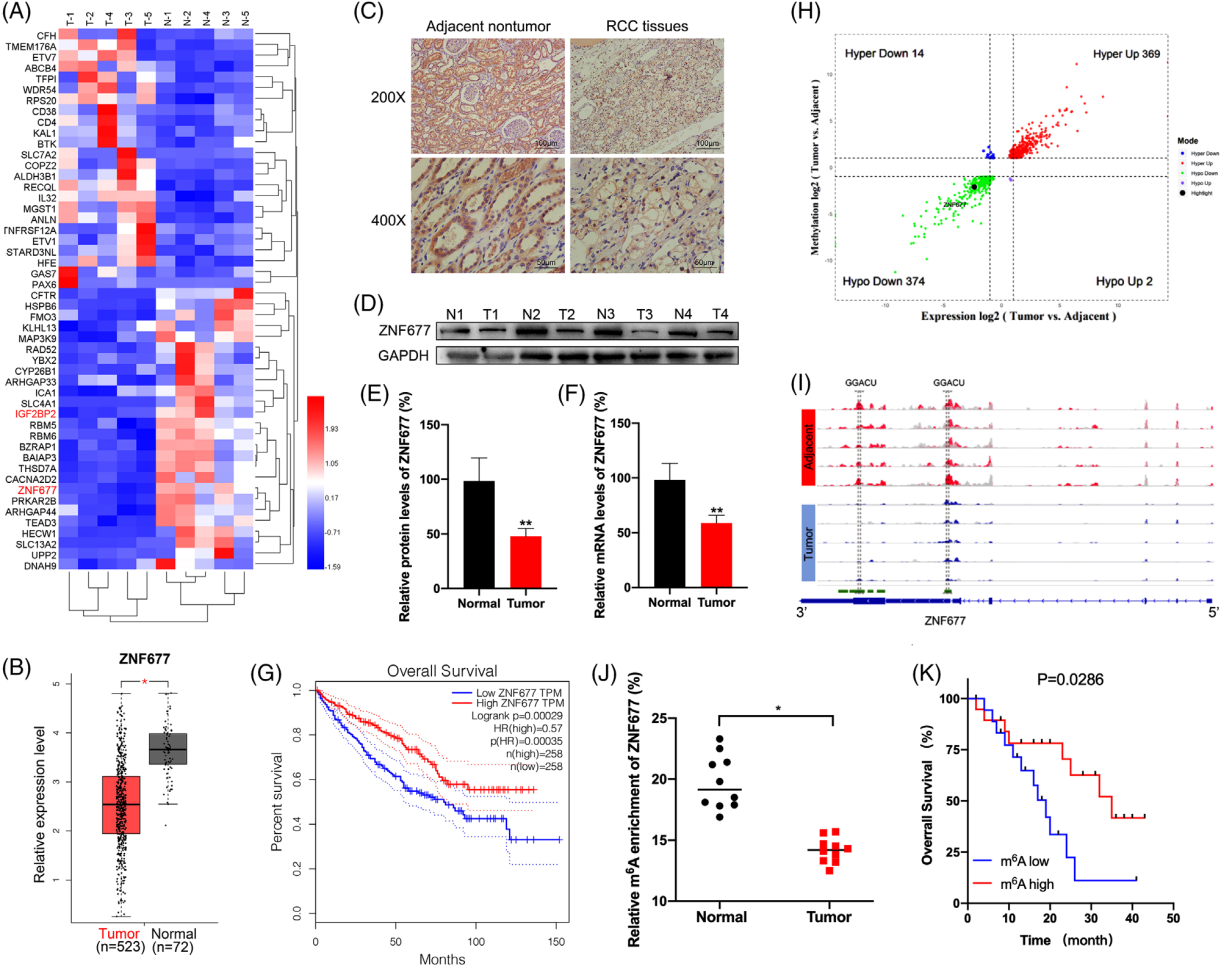
Downregulation of ZNF677 is associated with unfavourable prognosis and decreased m^6^A methylation modification levels in renal cell carcinoma (RCC). (A) Heatmap of differentially expressed genes in five pairs of matched RCC tissues and adjacent normal tissues by RNA‐seq. (B) Boxplot showing ZNF677 mRNA expression in 523 RCC tumour tissues (red plot) and 72 normal tissues (grey plot) (http://gepia2.cancer‐pku.cn). (C) Immunohistochemical analysis of ZNF677 performed on RCC tissues and adjacent normal tissues. (D) ZNF677 expression was determined by Western blot analysis in RCC tissues and their matched noncancerous tissues. GAPDH was used as loading control. (E) The quantitative illustration of the levels of ZNF677 protein in (D) was used for densitometry to measure the density of the corresponding bands on the Western blot analysis. (F) qRT‐PCR assay verified the expression of ZNF677 in matched RCC tissues and adjacent normal tissues. (G) Kaplan–Meier survival plot of RCC patients (*n* = 516) stratified by low (blue line) and high (red line) ZNF677 expression. (H) Four quadrant diagrams show the differentially methylated genes and differentially expressed genes in five pairs of matched RCC tissues and adjacent normal tissues detected by MeRIP‐seq and RNA‐seq. (I) Integrative Genome Viewer (IGV) software showed the m^6^A peaks within ZNF677 mRNA in five pairs of matched RCC tissues and adjacent normal tissues. (J) m^6^A enrichment on ZNF677 mRNA in 10 pairs of matched RCC tissues and adjacent normal tissues detected by MeRIP‐qPCR. (K) Kaplan–Meier survival analyses of the relationship between the levels of m^6^A of ZNF677 and survival time in RCC patients. *p < .05 or **p < .01 indicates a significant difference between the indicated groups

Previous studies have indicated that ZNF677 was downregulated by promoter methylation in non‐small lung cancer^26^ and thyroid cancer.^25^ However, a paucity of evidence had been found on the regulatory pathways controlling the ZNF677 expression levels in RCC. Herein, we first determined that the mRNA levels of ZNF677 were downregulated in five pairs RCC tissues compared to adjacent normal tissues from our RNA‐seq results (Figure 1A). This was further supported by The Cancer Genome Atlas (TCGA) database that ZNF677 expression in RCC was significantly lower than that in normal controls (Figure 1B and Figure S1A). Furthermore, we also analysed protein and mRNA expression of ZNF677 in 10 paired RCC and normal tissues by IHC, Western blot and quantitative reverse transcription polymerase chain reaction (RT‐qPCR) assays. As shown in Figure 1C–F and Figure S1C, ZNF677 was significantly downregulated in tumour tissues compared with adjacent normal tissues. Next, we attempted to evaluate the association of ZNF677 expression with patient survival using TCGA dataset. The results showed that decreased expression of ZNF677 was significantly associated with poor patient survival (Figure 1G and Figure S1B).

Evidently, m^6^A RNA methylation is a major inactivation mechanism of tumour suppressor genes in tumorigenesis.^27^, ^28^, ^29^ In order to investigate the roles of the m^6^A methylation modification‐associated genes in the progression of RCC, we used MeRIP‐seq to analyse the difference of the m^6^A methylation level in the five paired RCC tissues and adjacent normal tissues. MeRIP‐seq analysis revealed that there were 369 hypermethylated and upregulated (hyper‐up) genes, 374 hypomethylated and downregulated (hypo‐down) genes, 14 hypermethylated and downregulated (hyper‐down) genes and two hypomethylated and upregulated (hypo‐up) genes in RCC tissues compared to adjacent normal tissues (Figure 1H and Table S4). Especially, we found ZNF677 was one of hypo‐down gene in RCC tissues. In addition, our data detected that most of the m^6^A peaks were located at the 3′ untranslated region (3′‐UTR) and around the CDS‐3′UTR junction region of ZNF677 mRNA. Obviously, tumour tissues exhibited lower peak enrichment compared to the adjacent normal tissues (Figure 1I and Figure S1D–I). MeRIP‐qPCR assay confirmed that m^6^A level of ZNF677 was significantly lower in tumour tissues than normal tissues (Figure 1J). Finally, to further substantiate the survival significance of m^6^A methylation modification on ZNF677, we analysed the m^6^A levels of ZNF677 and its correlations with clinical behaviours of RCC patients. Kaplan–Meier survival analysis showed that low levels of m^6^A on ZNF677 were associated with a shorter overall survival (OS) in RCC patients (Figure 1K). Collectively, there was a correlation between ZNF677 expression/m^6^A status and OS.

### 2.2 m^6^A regulates mRNA stability and translation of ZNF677 in RCC cells

**FIGURE 2:**
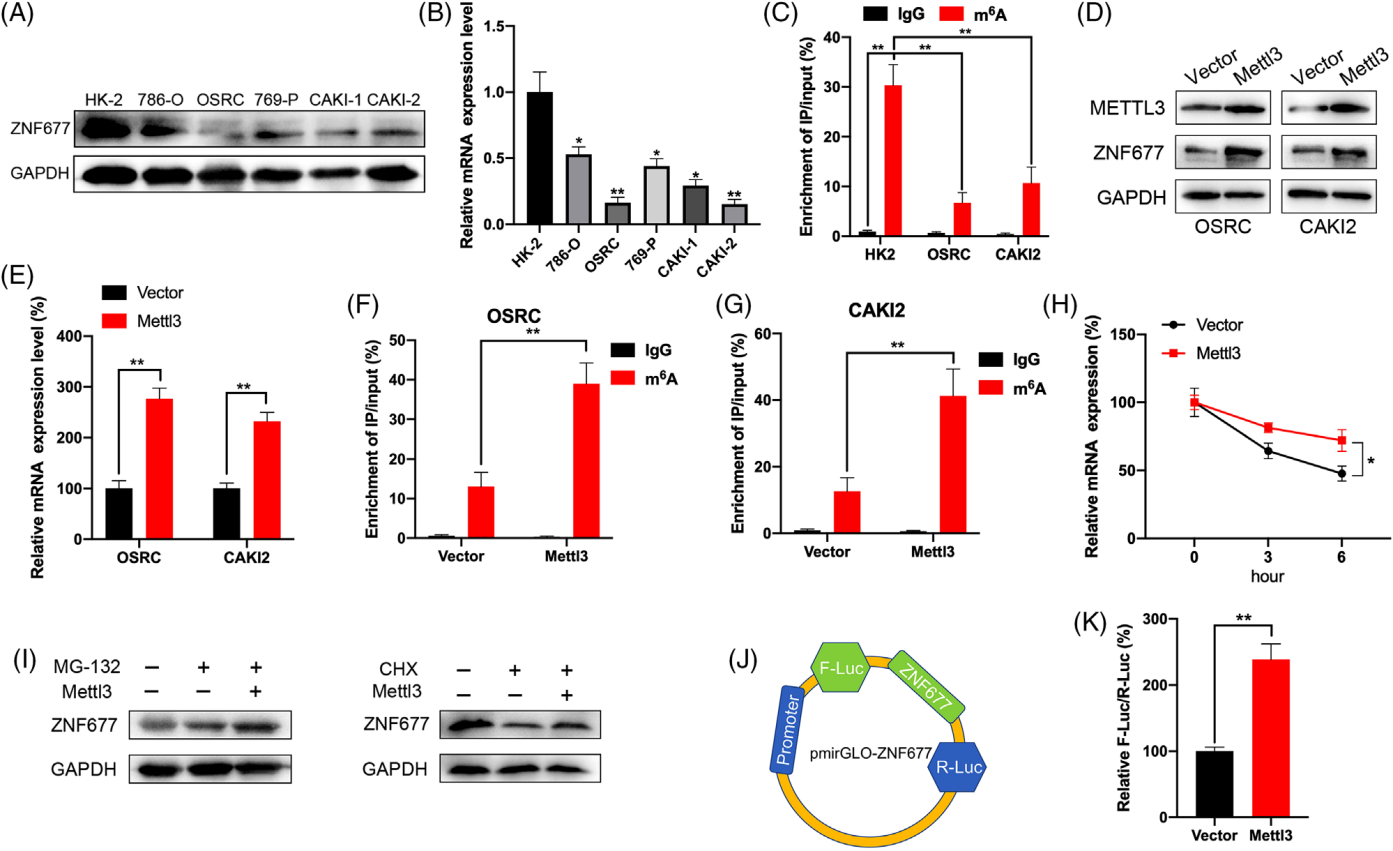
m^6^A regulates mRNA stability and translation of ZNF677 in renal cell carcinoma (RCC) cells. (A and B) Western blot (A) and RT‐qPCR (B) analysis of ZNF677 expression in different RCC cell lines (786‐O, OSRC, 769‐P, CAKI1, CAKI2) compared to normal epithelium cells of renal tubule HK2 cell. (C) MeRIP‐qPCR analysis of ZNF677 m^6^A levels in HK2, OSRC and CAKI2 cells. (D and E) OSRC and CAKI2 cells were transfected with vector control or Mettl3 construct for 24 h, the protein and mRNA expression levels of ZNF677 were measured by Western blot (D) and RT‐qPCR (E), respectively. (F and G) MeRIP‐qPCR analysis of ZNF677 m^6^A levels in control and overexpression of Mettl3 OSRC (F) and CAKI2 cells (G). (H) After treatment with Act‐D for the indicated times, the mRNA levels of ZNF677 were checked in control and Mettl3‐overexpressed OSRC cells. (I) OSRC cells were pretransfected with vector control or Mettl3 construct for 24 h and then further treated with CHX (10 μg/ml) or MG‐132 (5 μM) for 6 h, the expression of ZNF677 was detected by Western blot analysis. (J and K) Firefly (F‐Luc) values were normalised against Renilla luciferase levels, and ZNF677 translation efficiency was calculated for the pmirGLO‐ZNF677 reporter relative to pmirGLO in Mettl3 overexpression and control OSRC cells. NS, not significant; *p < .05 or **p < .01 indicates a significant difference between the indicated groups

To further investigate the potential mechanisms involved in m^6^A‐regulated expression of ZNF677, we first detected ZNF677 expression in a panel of RCC cell lines using Western blot and RT‐qPCR. Our data showed that ZNF677 expression was significantly decreased in all of five RCC cell lines compared to normal epithelium cells of renal tubule HK‐2 cell (Figure 2A,B). MeRIP‐qPCR confirmed that m^6^A enrichment of ZNF677 was also significantly decreased in OSRC and CAKI2 RCC cells compared to HK‐2 cell (Figure 2C). Next, we investigated the mechanisms via which m^6^A modification affects ZNF677 expression in RCC. Western blot and RT‐qPCR results showed that the protein and mRNA expressions of ZNF677 were increased in Mettl3‐overexpressing OSRC and CAKI2 cells (Figure 2D and Figure S2A,E). Further, MeRIP‐qPCR confirmed that overexpression of Mettl3 significantly promoted m^6^A antibody‐enriched ZNF677 mRNA in OSRC and CAKI2 cells (Figure 2F,G). In addition, we treated normal control and Mettl3‐overexpressing OSRC cells with Act‐D to block transcription. RNA stability assays suggested that overexpression of Mettl3 prolonged the half‐life of ZNF677 mRNA (Figure 2H). It indicated that m^6^A modification may delay the degradation of mRNA of ZNF677 in RCC cells.

We further investigated whether m^6^A can regulate the expression of ZNF677 beside mRNA stability. Normal control and Mettl3‐overexpressing OSRC cells were further treated with MG132 to inhibit proteasome activity or cycloheximide (CHX) to block protein translation. The data showed that in the presence of CHX, but not MG‐132, attenuated Metttl3 induced ZNF677 expression in OSRC cells (Figure 2I and Figure S2B), suggesting that Mettl3 might regulate the protein translation rather than protein stability/posttranslation modification of ZNF677. To confirm that m^6^A may regulate the translation of ZNF677, we constructed the pmirGLO‐ZNF677 luciferase reporter that contained ZNF677 cDNA in multiple cloning site (MCS) regions (Figure 2J). The subsequent results of dual‐luciferase assay indicated that the translational efficiency of ZNF677 was significantly greater in Mettl3‐overexpressing RCC cell than in the control group (Figure 2K). All these data suggested that m^6^A regulated the mRNA stability and translation of ZNF677 in RCC cells.

### 2.3 Methylation sites involved in m^6^A‐regulated ZNF677

**FIGURE 3:**
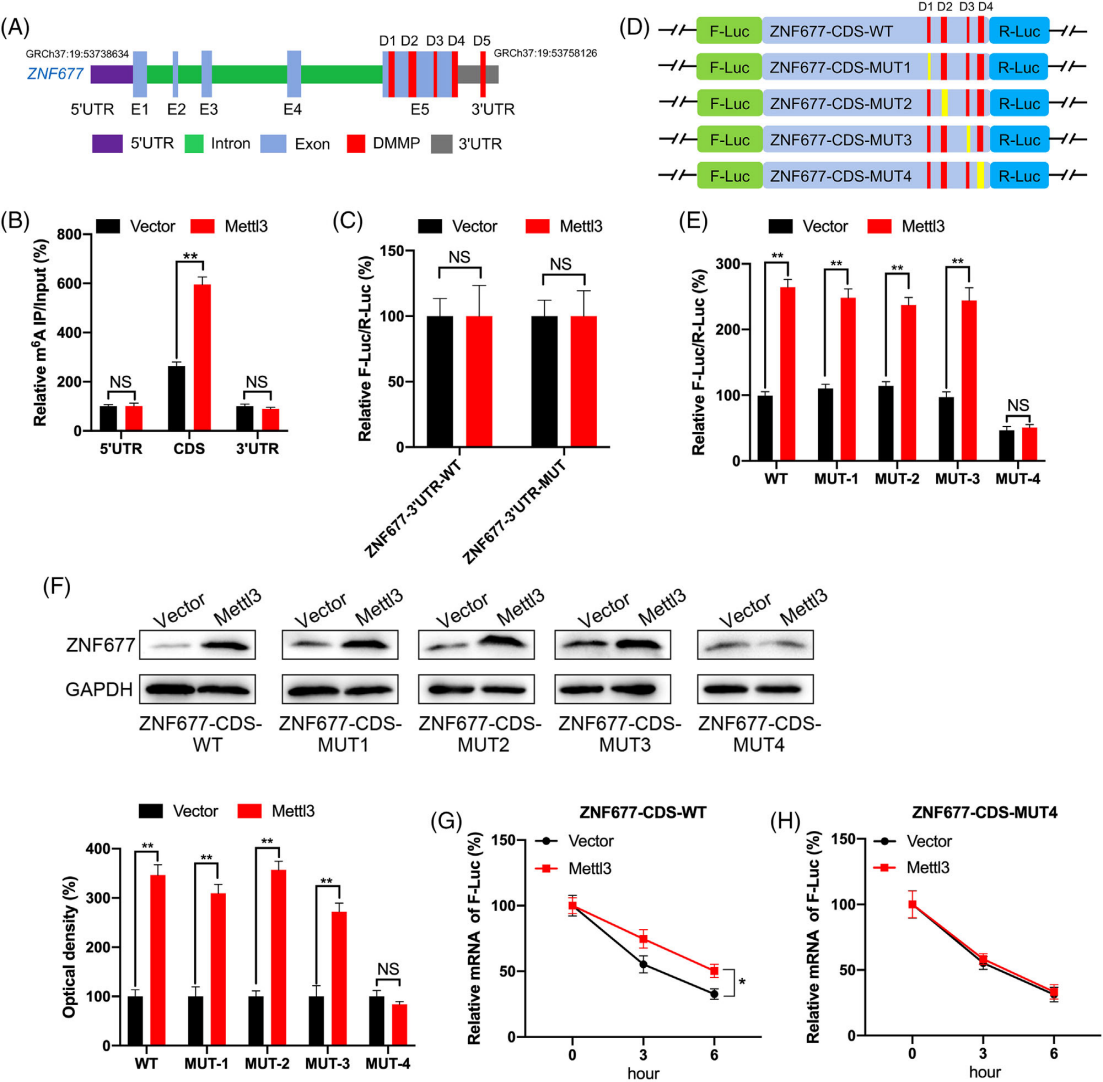
Methylation sites involved in m^6^A‐regulated ZNF677. (A) Schematic representation of positions of DMMPs within ZNF677 mRNA. (B) The m^6^A enrichment in 5′UTR, CDS or 3′UTR of ZNF677 in control or Mettl3 overexpression OSRC cells were analysed by MeRIP‐qPCR using fragmented RNA. (C) The relative luciferase activity of F‐Luc/R‐Luc of pmirGLO‐ZNF677‐3′UTR‐WT, or pmirGLO‐3′UTR‐MUT in control and Mettl3‐overexpressing OSRC cells. (D) Schematic representation of mutation in CDS of ZNF677 to investigate the m^6^A roles on ZNF677 expression. (E) The relative luciferase activity of F‐Luc/R‐Luc of pmirGLO‐ZNF677‐CDS‐WT, or pmirGLO‐ZNF677‐CDS‐MUT‐1/‐2/‐3/‐4 in control and Mettl3‐overexpressing OSRC cells. (F) Western blot analysis of ZNF677 expression in OSRC cells co‐expressing exogenous Mettl3 and pmirGLO‐ZNF677‐CDS‐WT or pmirGLO‐ZNF677‐CDS‐MUTs. (G and H) pmirGLO‐ZNF677‐CDS‐WT (G) or pmirGLO‐ZNF677‐CDS‐MUT4 (H) was transfected into control or Mettl3‐overexpressing OSRC cells for 24 h and then further treated with Act‐D for the indicated times. The mRNA of ZNF677 was checked by RT‐qPCR. NS, not significant; *p < .05 or **p < .01 indicates a significant difference between the indicated groups

MeRIP‐seq data showed that there were four differentially methylated m^6^A peaks (DMMP) in CDS region and one DMMP in 3′UTR region of ZNF677 mRNA (Figure 1I and Figure 3A). To characterise m^6^A methylation in ZNF677 mRNA, fragmented RNA isolated from OSRC cell was immunoprecipitated with m^6^A antibody. MeRIP‐qPCR showed that the highest level of m^6^A methylation was observed in the CDS, followed by the 3′UTR and 5′UTR (Figure 3B). Accordingly, increased m^6^A enrichment was observed in the ZNF677 CDS in Mettl3‐overexpressing cells, indicating that m^6^A modification is more dynamic in the CDS than in the 3′UTR region (Figure 3B).

To explore the potential roles of m^6^A methylation in the ZNF677 3′UTR region, which demonstrated m^6^A methylation at DMMP5, we performed a luciferase assay in OSRC cell using reporters containing ZNF677–3′UTR‐WT or ‐MUT. Compared to ZNF677–3′UTR‐WT, the translational activity of ZNF677–3′UTR‐MUT was similar between the control and Mettl3‐overexpressing groups (Figure 3C). Together, these results indicate that the m^6^A modification, which promotes ZNF677 mRNA stability and translation, does not correlate with m^6^A methylation level at DMMP5 in the 3′UTR.

Therefore, we examined whether m^6^A methylation in the CDS promotes the translation of ZNF677. First, we constructed a ZNF677 CDS expression plasmid and mutated the m^6^A motif in the coding region of ZNF677 as follows: ZNF677‐CDS‐MUTs series: ZNF677‐CDS‐MUT1, ZNF677‐CDS‐MUT2, ZNF677‐CDS‐MUT3 and ZNF677‐CDS‐MUT4 (containing four DMMPs, of which only one was mutated to create ZNF677‐CDS‐MUT1/2/3/4) (Figure 3D). The dual‐luciferase assay showed that the translation efficiency of ZNF677‐CDS‐WT in Mettl3‐overexpressing OSRC cell was significantly higher than that in control cell (Figure 3E). Compared to the control group, ZNF677‐CDS‐MUT1/2/3, while not ZNF677‐CDS‐MUT4, resulted in an upregulation of translation efficiency of F‐Luc in Mettl3‐overexpressing cells (Figure 3E). Notably, Western blot results showed that promotion of Mettl3‐activated ZNF677 expression was attenuated using ZNF677‐CDS‐MUT4, compared to that of ZNF677‐CDS‐WT or ZNF677‐CDS‐MUT1/2/3 (Figure 3F). This was further confirmed by the results that the mRNA stability of ZNF677‐CDS‐WT in Mettl3‐overexpressing cell was greater than that in control cells (Figure 3G), while ZNF677‐CDS‐MUT4 can abolish the difference of mRNA half‐lives between control and Mettl3‐overexpressing cells (Figure 3H). Together, our data suggested that Mettl3 site‐specific m^6^A modification in ZNF677 CDS is responsible for the ZNF677 mRNA stability and translation.

### 2.4 Factors involved in m^6^A‐regulated expression of ZNF677

**FIGURE 4:**
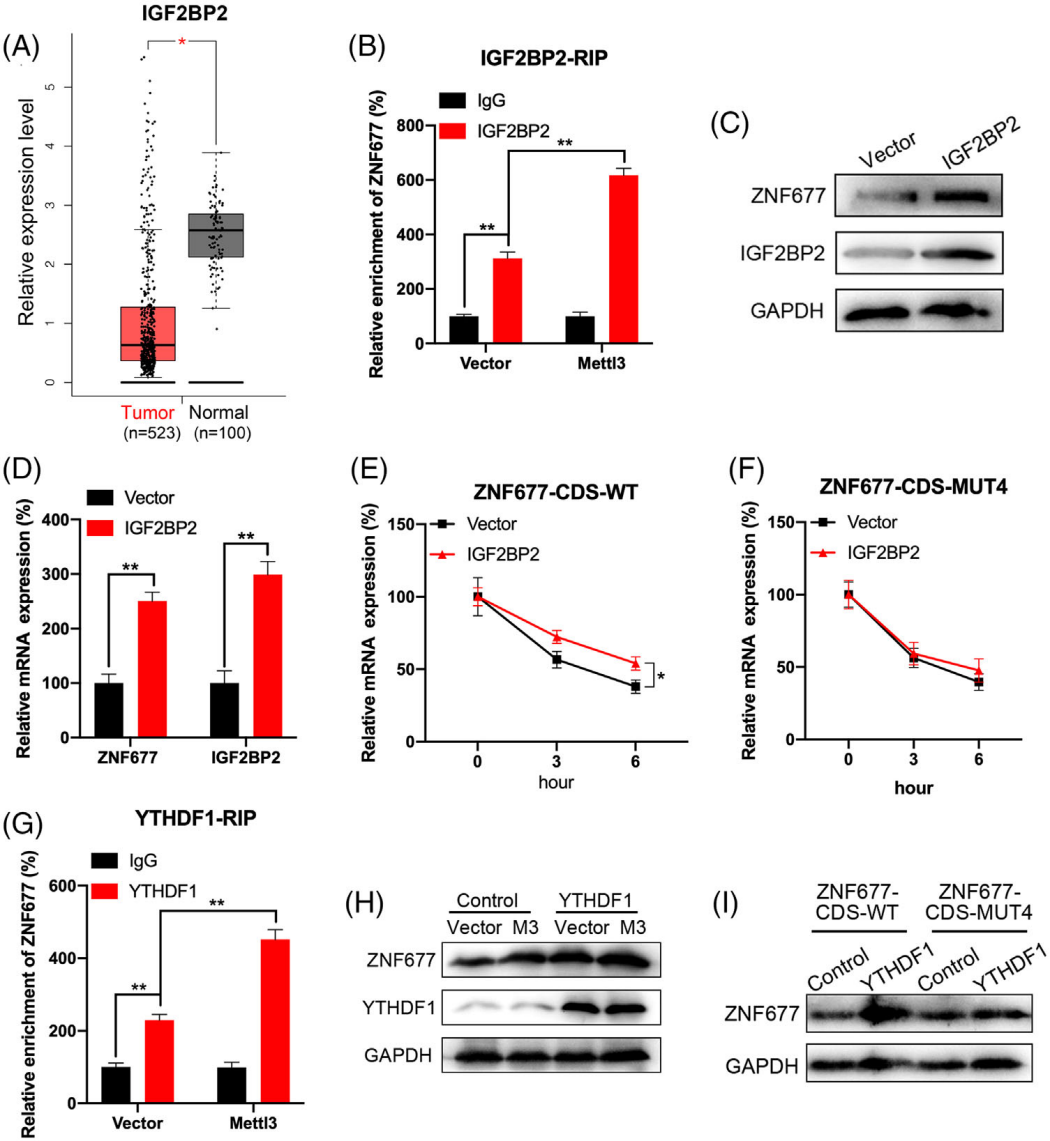
Factors involved in m^6^A‐regulated expression of ZNF677. (A) Results based on TCGA and GEPIA database (http://gepia.cancer‐pku.cn/index) showed the expression level of IGF2BP2 in renal cell carcinoma (RCC) tumours and normal tissues. (B) IGF2BP2 RIP‐qPCR analysis of ZNF677 mRNA in control or Mettl3‐overexpressing OSRC cells. (C and D) Western blot and RT‐qPCR analysis of ZNF677 and IGF2BP2 protein (C) and mRNA (D) expression in OSRC cells transfected with control vector or IGF2BP2 construct. (E and F) OSRC cells were transfected with control vector, IGF2BP2 construct, pmirGLO‐ZNF677‐CDS‐WT (E) or pmirGLO‐ZNF677‐CDS‐MUT4 (F) for 24 h and then further treated with Act‐D for the indicated times. The mRNA of ZNF677 was checked by RT‐qPCR. (G) YTHDF1 RIP‐qPCR analysis of ZNF677 mRNA in control or Mettl3‐overexpressing OSRC cells. (H) Control or Mettl3‐overexpressing OSRC cells were transfected with control vector or YTHDF1 construct for 24 h, the expression of ZNF677 was checked by Western blot analysis. (I) OSRC cells were transfected with control vector, YTHDF1 construct, pmirGLO‐ZNF677‐CDS‐WT and pmirGLO‐ZNF677‐CDS‐MUT4 for 24 h, the expression of ZNF677 was checked by Western blot analysis. NS, not significant; *p < .05 or **p < .01 indicates a significant difference between the indicated groups

Mechanisms responsible for m^6^A‐regulated mRNA stability and translation were further investigated. It has been revealed that m^6^A modification may regulate the mRNA stability via readers, including YTHDF2, YTHDF3 and IGF2BP1∼3.^30^, ^31^ Combined with our data, the RNA‐seq results revealed that the expression of IGF2BP2 was significantly decreased in RCC tumour tissues (Figure 1A). Additionally, IGF2BP2 was found to be evidently downregulated in RCC tumour samples (523 samples) compared with normal samples (100 samples) from TCGA database (Figure 4A). Therefore, we verified whether IGF2BP2 was m^6^A‐dependent in a manner that enhances the mRNA stability of ZNF677. First, we detected the enrichment of IGF2BP2 binding to ZNF677 m^6^A modification sites by RIP‐qPCR assay. Results showed that IGF2BP2 can significantly bind with ZNF677 mRNA in OSRC cell (Figure 4B). Further, the binding between IGF2BP2 and ZNF677 was significantly increased in Mettl3‐overexpressing cell as compared with that in control cell (Figure 4B). We therefore overexpressed IGF2BP2 in OSRC cell. Results showed that IGF2BP2 can promote the protein and mRNA levels of ZNF677 in OSRC cell (Figure 4C,D and Figure S2C). Further, IGF2BP2 can significantly increase the mRNA stability of ZNF677‐CDS‐WT (Figure 4E), while this effect was attenuated for ZNF677‐CDS‐MUT4 (Figure 4F). These data suggested that IGF2BP2 was involved in m^6^A methylation modification and regulated ZNF677 mRNA stability.

YTHDF1 can recognise m^6^A‐methylated mRNA and promote the translation of its targets.^32^ RIP‐qPCR was used to verify whether YTHDF1 participates in m^6^A methylation of ZNF677 mRNA. Results showed that YTHDF1 interacted with ZNF677 mRNA remarkably, while this interaction was significantly increased in Mettl3‐overexpressing cells (Figure 4G). To confirm the roles of YTHDF1 in m^6^A‐regulated ZNF677 expression, we overexpressed YTHDF1 in OSRC cell. Our data showed that YTHDF1 can increase the protein expression level of ZNF677 and promote upregulation of Mettl3‐activated expression of ZNF677 in OSRC cells (Figure 4H and Figure S2D). Further, overexpression of YTHDF1 can increase the protein expression of ZNF677‐CDS‐WT, while this effect was attenuated for ZNF677‐CDS‐MUT4 (Figure 4I and Figure S2E). In summary, these data revealed that IGF2BP2 affected the mRNA stability of ZNF677, and YTHDF1 participates in the translation ZNF677 by participating in the ZNF677 m^6^A modification.

### 2.5 Targeting m^6^A methylation of ZNF677 by CRISPR/dCas13b‐METTL3 to regulate RCC cells proliferation and apoptosis

**FIGURE 5:**
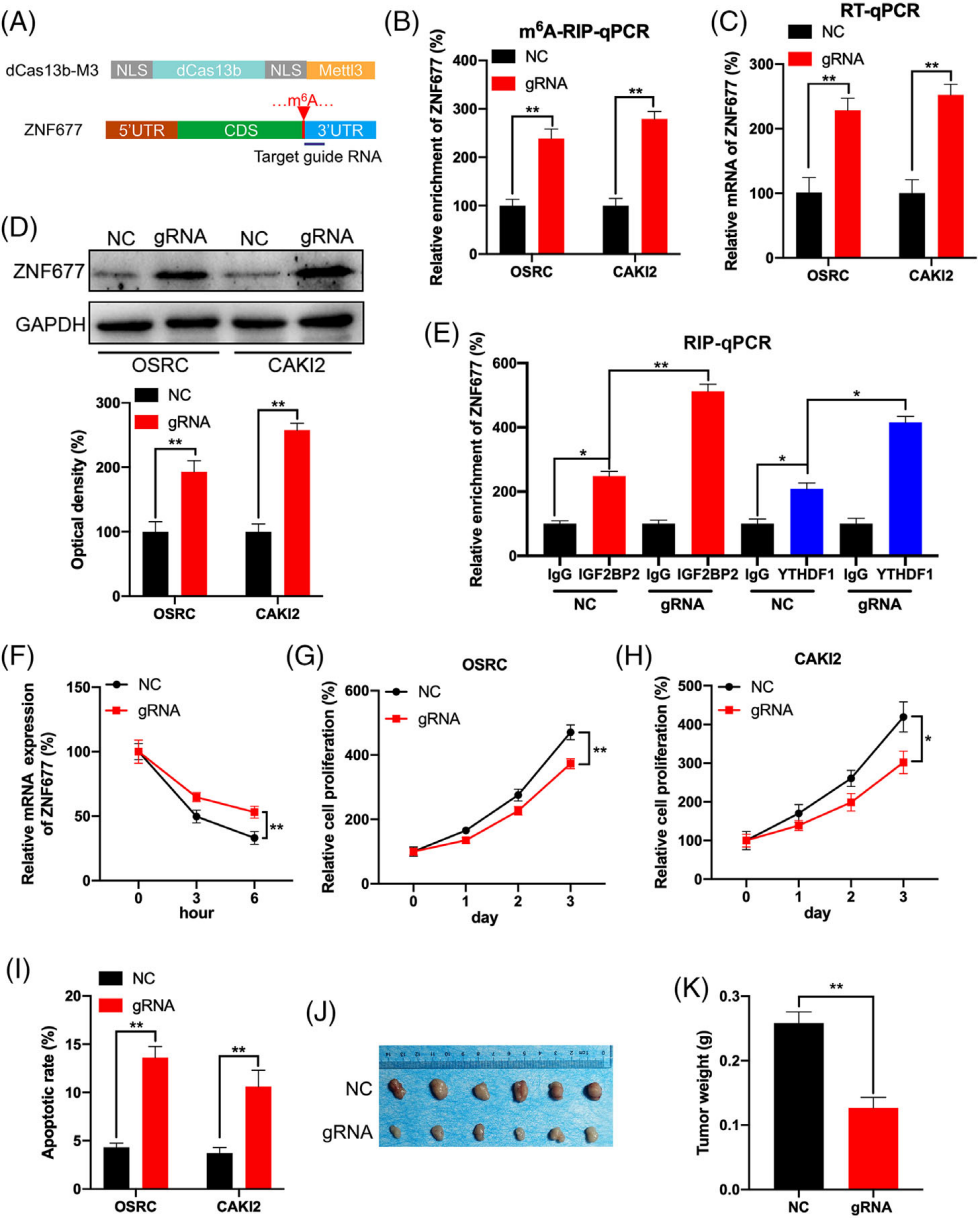
Targeting m^6^A methylation of ZNF677 by CRISPR/dCas13b‐METTL3 to regulate renal cell carcinoma (RCC) cells proliferation and apoptosis. (A) Schematic representation of positions of m^6^A site within ZNF677 mRNA and the regions targeted by target guide RNA. (B–D) The m^6^A (B), mRNA (C) and protein level (D) of ZNF677 in OSRC and CAKI2 cells transfected with dCas13b‐METTL3 combined with gRNA control or gRNA for ZNF677, respectively, for 24 h. (E) RIP‐qPCR analysis of ZNF677 mRNA in OSRC cells transfected with dCas13b‐METTL3 combined with gRNA control or gRNA for ZNF677 for 24 h by use of antibodies against IGF2BP2 and YTHDF1, respectively. (F) OSRC cells were transfected with gRNA control, gRNA for ZNF677 and dCas13b‐METTL3 for 24 h and then further treated with Act‐D for the indicated times. The mRNA of ZNF677 was checked by RT‐qPCR. (G and H) CCK8 assay analysis of cell proliferation of OSRC (G) and CAKI2 (H) cells transfected with dCas13b‐METTL3 combined with gRNA control or gRNA for ZNF677 for 24 h. (I) The cell apoptosis of OSRC and CAKI2 cells transfected with dCas13b‐METTL3 combined with gRNA control or gRNA for ZNF677 for 24 h detected by caspase‐3 ELISA kit. (J and K) The tumour weights of OSRC cells stably transfected with dCas13b‐METTL3 combined with gRNA control or gRNA for ZNF677. NS, not significant; *p < .05 or **p < .01 indicates a significant difference between the indicated groups

We then specifically methylated the m^6^A of ZNF677 by fusing the catalytically dead type VI‐B Cas13 enzyme with the m^6^A methylase METTL3 (dCas13b‐M3).^33^ Targeted guide RNA at distinct position around the m^6^A site was designed to target the mRNA of ZNF677 (Figure 5A). First, the dCas13b‐M3‐induced m^6^A methylation of ZNF677 was confirmed by MeRIP‐qPCR in OSRC and CAKI2 RCC cells (Figure 5B). Next, our results showed that dCas13b‐M3 targeting ZNF677 led to a significant upregulation of ZNF677 mRNA (Figure 5C) and protein levels (Figure 5D) in OSRC and CAKI2 cells. This might be due to that dCas13b‐M3 with gRNA for ZNF677 can significantly increase the binding of ZNF677 mRNA with IGF2BP2 and YTHDF1 (Figure 5E). To investigate whether m^6^A‐mediated mRNA stability of ZNF677 was related to the dCas13b‐M3‐induced upregulation of ZNF677, we compared the effects of dCas13b‐M3 with control or gRNA on ZNF677 mRNA half‐life. Results showed that targeted methylation of ZNF677 can significantly stabilise its mRNA (Figure 5F), indicating that dCas13b‐M3 increased the mRNA stability via methylation of m^6^A at CDS in the case of ZNF677.

To further investigate that dCas13b‐M3‐targeting ZNF677 can modulate RCC cells homeostasis, we detected the cell proliferation and apoptosis after transfected with control or gRNA for ZNF677 combined with dCas13b‐M3 in OSRC and CAKI2 RCC cells. Our data showed that gRNA for ZNF677 can significantly decrease the cell proliferation and promote cell apoptosis as compared with that of non‐targeted control gRNA combined with dCas13b‐M3 in OSRC and CAKI2 cells (Figure 5G–I; Figures S3A and S5A). Control or gRNA for ZNF677 combined with dCas13b‐M3 stable OSRC cells were used to establish xenografts. Consistently, xenograft model confirmed that m^6^A methylation of ZNF677 can inhibit tumour growth in vivo (Figure 5J,K).

### 2.6 ZNF677 transcriptionally regulates expression of CDKN3

**FIGURE 6:**
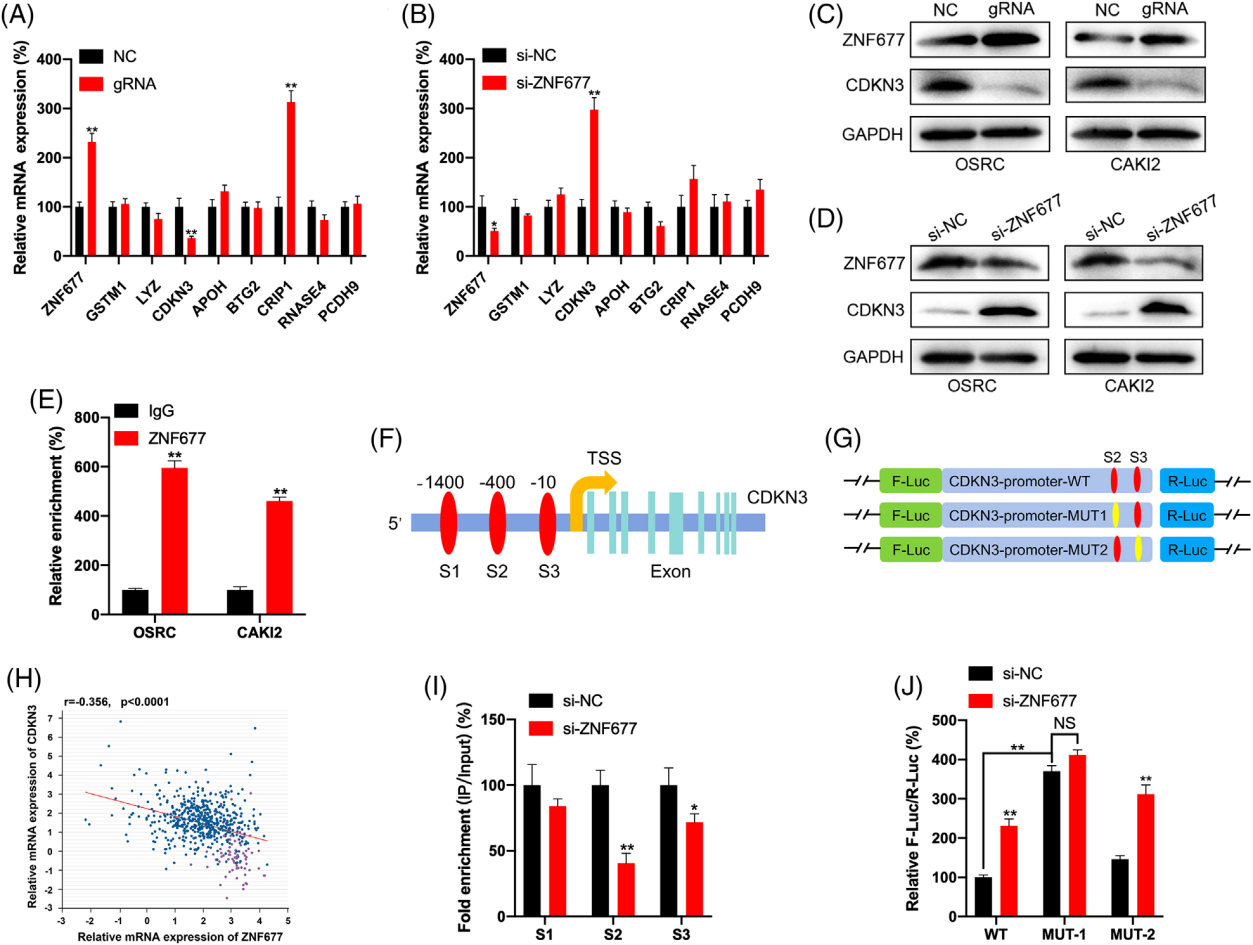
ZNF677 transcriptionally regulates expression of CDKN3. (A) The mRNA expression of potential target genes of ZNF677 in OSRC cells transfected with dCas13b‐METTL3 combined with gRNA control or gRNA for ZNF677 for 24 h were checked by RT‐qPCR analysis. (B) The mRNA expression of potential target genes of ZNF677 in OSRC cells transfected with siRNA control or siRNA for ZNF677 for 24 h were checked by RT‐qPCR analysis. (C) The protein expressions of ZNF677 and CDKN3 in OSRC and CAKI2 cells transfected with dCas13b‐METTL3 combined with gRNA control or gRNA for ZNF677 for 24 h were checked by Western blot analysis. (D) The protein expressions of ZNF677 and CDKN3 in OSRC and CAKI2 cells transfected with siRNA control or siRNA for ZNF677 for 24 h were checked by Western blot analysis. (E) The binding between ZNF677 and promoter of CDKN3 was checked by ChIP‐qPCR using IgG or ZNF677 antibody. (F) Schematic representation of the potential binding sites 1, 2 and 3 between ZNF677 and the promoter of CDKN3. (G) Binding between ZNF677 and the promoter of CDKN3 at the potential binding sites 1, 2 and 3 in OSRC cells transfected with siRNA control or siRNA for ZNF677 for 24 h was checked by ChIP‐qPCR. (H) Schematic representation of the mutated CDKN3 promoter reporter to investigate the role of ZNF677 in CDKN3 expression. (I) OSRC cells were co‐transfected with CDKN3‐promoter‐WT, CDKN3‐promoter‐MUT1, CDKN3‐promoter‐MUT2 and si‐NC or si‐ZNF677 for 24 h. Results were presented as the ratio between the activity of the reporter plasmid. (J) Correlation between ZNF677 and CDKN3 in renal cell carcinoma (RCC) patients from ChIPBase database. Data are presented as the mean ± SD from three independent experiments. NS, not significant; *p < .05 or **p < .01 indicates a significant difference between the indicated groups

To explore molecular mechanisms underlying tumour‐suppressive effects of ZNF677 in RCC, we first tested the effect of ZNF677 on the expression of downstream targets. A previous study has identified a number of genes negatively regulated by ZNF677 in NSCLC cells.^26^ Of them, we identified eight genes that showed a significantly aberrant expression in RCC samples compared to normal samples (Figure S4A). We further determined transcriptional regulation of these genes by ZNF677 in OSRC cells. As shown in Figure 6A–D and Figure S4B–C, upregulation of ZNF677 dramatically inhibited the expression of CDKN3 at both mRNA and protein levels, while ZNF677 knockdown significantly activated the expression of CDKN3. Chromatin immunoprecipitation (ChIP)‐qPCR assays demonstrated that ZNF677 had a significant enrichment of CDKN3 promoter over normal immunoglobulin G (IgG) control in both OSRC and CAKI2 cells (Figure 6E), indicating a direct binding between ZNF677 and CDKN3 promoter. ChIPBase data showed that there were three ZNF677 potential binding sites in the promoter within 1.5 kb upstream of CDKN3 (Figure 6F). Next, ChIP‐qPCR showed that binding of ZNF677 to the potential binding sites 2 and 3 was much less than to the site 1 after downregulation of ZNF677 in OSRC cells (Figure 6I). Therefore, we mutated the potential binding sites 2 and 3 of promoter reporter of CDKN3 to generate the CDKN3‐promoter‐MUT1 or CDKN3‐promoter‐MUT2 (Figure 6G). Our data showed that si‐ZNF677 can significantly increase luciferase levels of CDKN3‐promoter‐WT and CDKN3‐promoter‐MUT2, while the promotion effect of si‐ZNF677 was attenuated for CDKN3‐promoter‐MUT1 (Figure 6J). Further, the expression of ZNF677 was significantly and negatively correlated with the expression of CDKN3 in clinical RCC patients from ChIPBase (Figure 6H). All these data suggested that ZNF677 might be responsible for the regulation of CDKN3 in RCC via binding to its promoter‐proximal site to inhibit its transcription.

### 2.7 CDKN3 knockdown inhibits RCC cells proliferation and promotes cell apoptosis and rescues the antitumour phenotype impaired by ZNF677 deficiency

**FIGURE 7:**
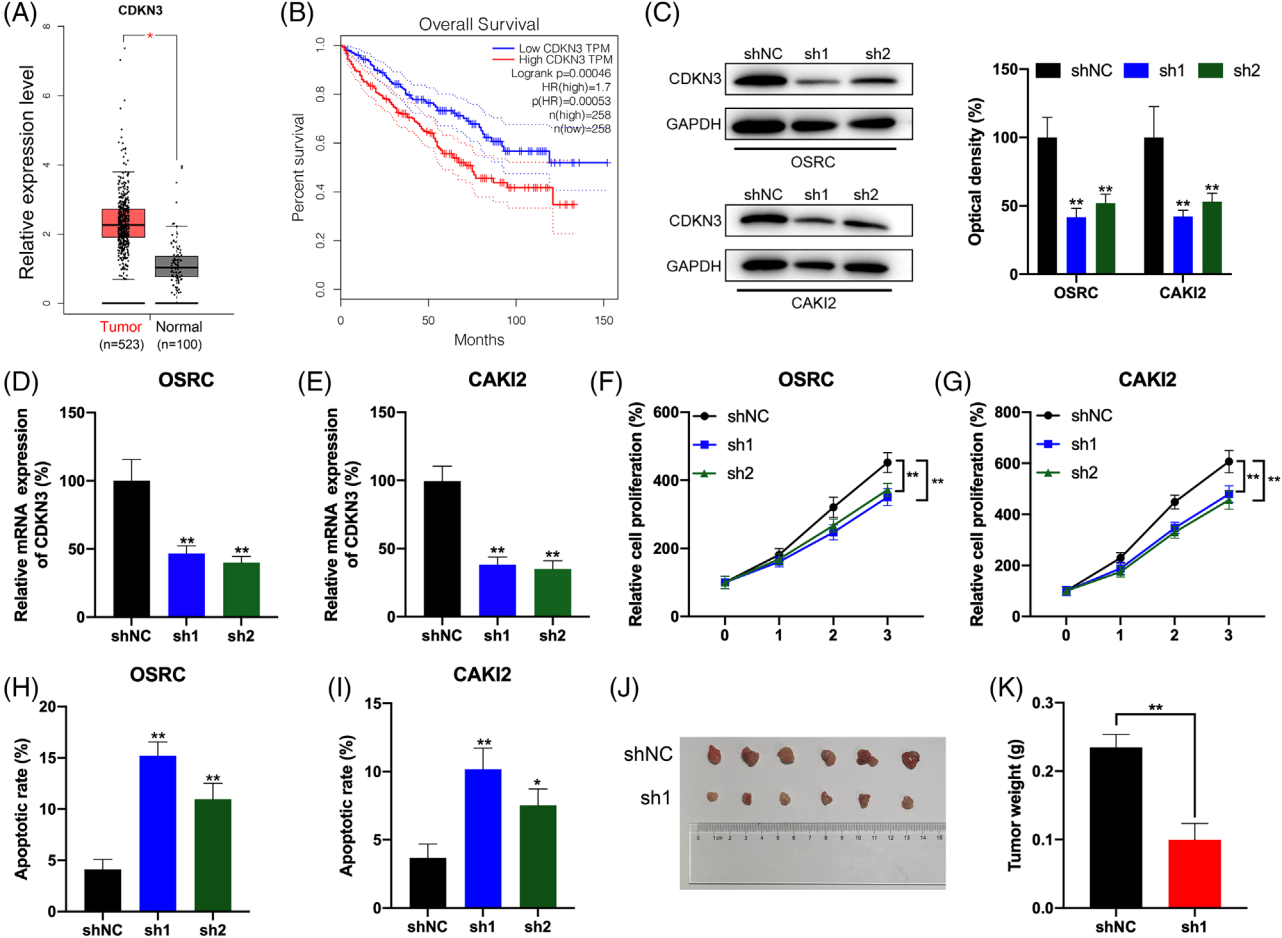
CDKN3 knockdown inhibits renal cell carcinoma (RCC) cells proliferation and promotes cells apoptosis. (A) Boxplot showing CDKN3 mRNA levels in RCC tumours (red box) versus normal renal tissues (grey box) from TCGA database. (B) Kaplan–Meier survival plot of RCC patients (*n* = 516) stratified by low (blue line) and high (red line) CDKN3 expression. (C) Western blot analysis to measure CDKN3 protein levels in OSRC and CAKI2 cells transfected with shRNA control (shNC) and shRNA for CDKN3 (sh1 or sh2). (D and E) RT‐qPCR analysis to measure CDKN3 mRNA levels in OSRC (D) and CAKI2 (E) cells transfected with shNC and shRNA for CDKN3 (sh1 or sh2). (F and G) The cell proliferation of OSRC (F) and CAKI2 (G) cells was transfected with shNC and shRNA for CDKN3 (sh1 or sh2) detected by CCK8 assay. (H and I) The cell apoptosis of OSRC (H) and CAKI2 (I) cells transfected with shNC and shRNA for CDKN3 (sh1 or sh2) was detected by caspase‐3 ELISA kit. (J and K) The tumour weights of OSRC cells stably transfected with shNC or sh1 for CDKN3. *p < .05 or **p < .01 indicates a significant difference between the indicated groups

In order to explore the potential function of CDKN3 in RCC, we analysed the expression of CDKN3 in RCC tissues and noncancerous normal tissues, and found that CDKN3 is frequently elevated in tumour tissues from the TCGA database (Figure 7A and Figure S6). The Kaplan–Meier survival analysis displayed that increased expression of CDKN3 is significantly correlated with worse OS in RCC patients (Figure 7B). Then we synthesised CDKN3‐specific shRNA to obstruct the expression of endogenous CDKN3 in OSRC and CAKI2 cells. CDKN3 protein expression was reduced after shCDKN3 transfection (Figure 7C). Similarly, RT‐qPCR results were confirmed at the mRNA level (Figure 7D,E). Cell Counting Kit‐8 (CCK‐8) assays showed that CDKN3 knockdown significantly repressed cell proliferation of OSRC and CAKI2 cells (Figure 7F,G and Figure S3B). After knocking down CDKN3, the apoptosis of OSRC and CAKI2 cells was obviously increased (Figure 7H,I and Figure S5B). To further evaluate the effects of CDKN3 on the regulation of RCC cells progression in vivo, RCC cells were subcutaneously injected into mice. As expected, when OSRC cells with downregulated CDKN3 expression were injected, the xenograft tumour size was significantly reduced compared to the control group (Figure 7J). And the tumour weights in the CDKN3 knockdown group were significantly decreased compared to those in the control group (Figure 7K).

**FIGURE 8:**
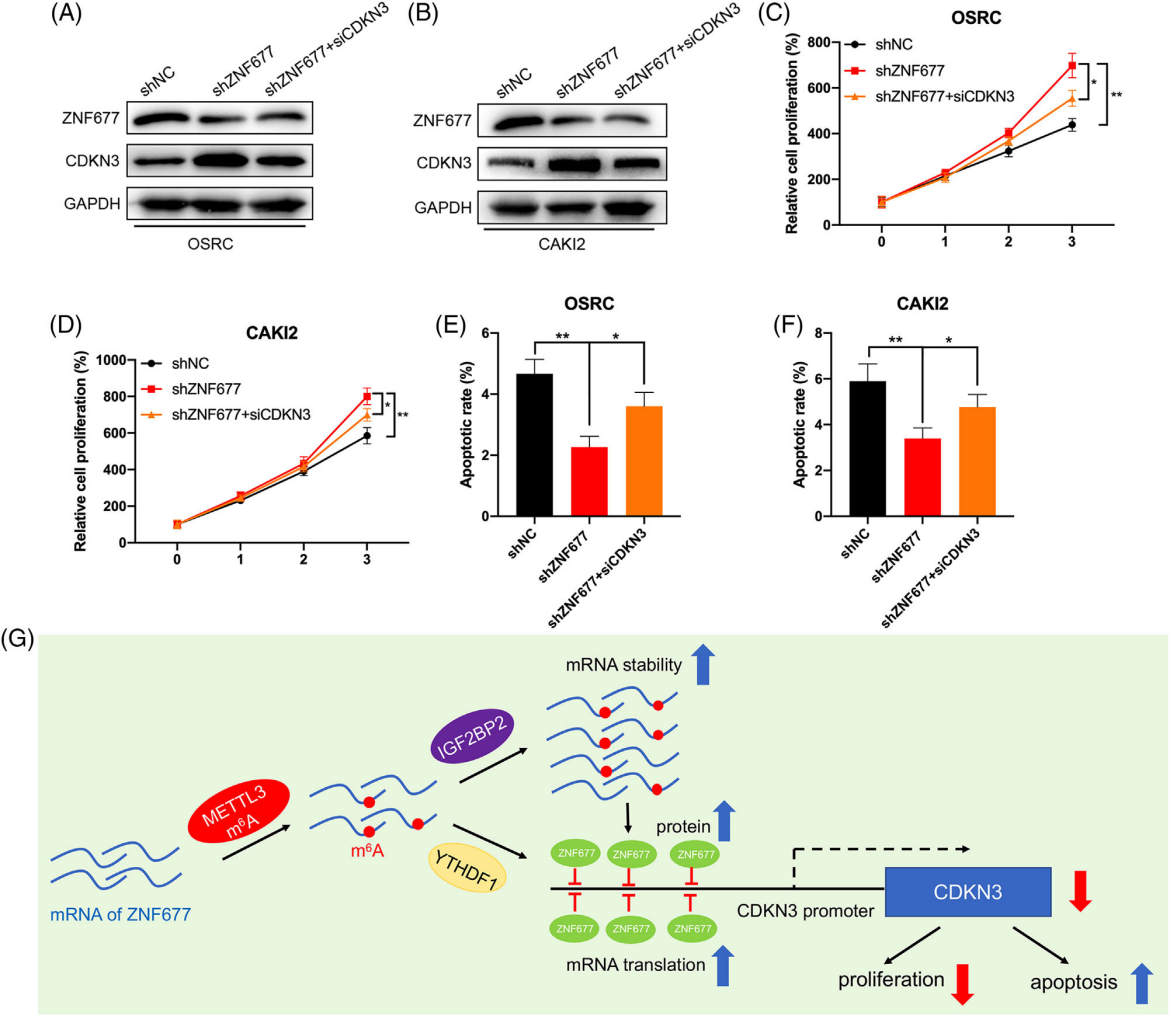
CDKN3 knockdown rescues the antitumour phenotype impaired by ZNF677 deficiency. (A and B) Western blot was performed to investigate the expression of ZNF677 and CDKN3 in OSRC (A) and CAKI2 (B) cells after transfection with shZNF677 and/or siCDKN3. (C and D) The cell proliferation of OSRC (C) and CAKI2 (D) cells transfected with shZNF677 and/or siCDKN3 was detected by CCK8 assay. (E and F) The cell apoptosis of OSRC (E) and CAKI2 (F) cells transfected with shZNF677 and/or siCDKN3 was detected by caspase‐3 ELISA kit. (G) The working model for the regulation of m^6^A‐dependent turnover of ZNF677 mRNA by interacting with the m^6^A readers IGF2BP2 and YTHDF1, and ZNF677 binds to the promoter of its target CDKN3 to regulate cellular activities. *p < .05 or **p < .01 indicates a significant difference between the indicated groups

To further determine the roles of CDKN3 in tumour‐suppressive effect of ZNF677 on RCC cells, we knocked down CDKN3 in OSRC and CAKI2 cells with ZNF677 deficiency. Western blot results revealed that the ablation of CDKN3 could partially neutralise the increased effects of inhibited ZNF677 on the expression of CDKN3 in OSRC and CAKI2 cells (Figure 8A,B and Figure S5D,E). Moreover, CDKN3 knockdown partially reversed the effects of ZNF677 depletion on the proliferation and apoptosis of OSRC and CAKI2 cells (Figure 8C–E; Figures S3C and S5C).

Altogether, based on the above findings, we propose a model to explore molecular mechanisms of ZNF677 inhibiting RCC tumorigenesis (Figure 8G). In RCC cells, ZNF677 binds to the promoter of its target CDKN3 to regulate cellular activities, and is modified through an m^6^A‐dependent turnover mechanism by interacting with the m^6^A readers IGF2BP2 and YTHDF1.

## 3 DISCUSSION

m^6^A is identified as a dynamic and reversible RNA modification in eukaryotes, due to the ‘writer’ (methyltransferase) and ‘eraser’ (demethylase) proteins. It has been reported that m^6^A modification takes part in many cellular activities and reaction, including heat shock,^34^ ultraviolet light,^35^ hypoxic stress^36^ and oxidative stress.^37^ Despite there are numbers of evidences confirming that m^6^A modification could promote the development of tumours, their roles in RCC have been less reported. ZNF677 belongs to the zinc finger protein family, which possesses transcription factor activity by binding sequence‐specific DNA. Previous studies have reported its downregulation by promoter methylation in various tumours.^25^, ^26^, ^37^, ^38^ However, whether m^6^A methylation modification could influence ZNF677 expression to alter epigenetic remodelling or contribute to the malignant features of RCC is unclear and worthy of investigation.

In this study, we provided strong evidences supporting that ZNF677 is a potent tumour suppressor in RCC. First, we identified many differentially methylated genes in RCC tumour tissues versus tumour‐adjacent normal tissues based on MeRIP‐seq technology and found that ZNF677 was frequently downregulated in RCC tumours. And ZNF677 downregulation was associated with poor patient outcomes. Moreover, our data showed inactivation of ZNF677 might be caused by significantly decreased enrichment of m^6^A methylation modification in its CDS and 3′UTR regions in tumour tissues. Overexpression of Mettl3 can increase the m^6^A and expression level of ZNF677 by positively regulating the mRNA stability and translation of ZNF677.

The m^6^A modification can regulate nearly all stages in the lifecycle of RNA, such as RNA processing, nuclear export and translation modulation.^39^, ^40^ Our data showed that m^6^A in CDS, rather than 3′UTR, positively regulated m^6^A methylation modification of ZNF677 by luciferase reporter system. In addition, IGF2BP2 was involved in m^6^A‐regulated mRNA stability of ZNF677, while YTHDF1 is likely involved in the m^6^A‐regulated protein translation in RCC cells. Consistently, another study indicated that IGF2BPs can bind the GG(m^6^A)C sequence of mRNA to promote the stability and storage of their target mRNAs.^30^ And methylation of CDS of Snail, which recruited the YTHDF1 and eEF‐2, can trigger its translation elongation and cancer metastasis.^41^ Thus, our data provided a totally new insight into the function of IGF2BP2 and YTHDF1 in RCC via regulating the mRNA stability and translation of ZNF677, respectively.

We further specifically methylated the m^6^A of ZNF677 mRNA by use of CRIPSR/dCas13b‐METTL3.^33^ The system resulted in about two‐fold increase in methylation and significantly increased the expression of ZNF677. In RCC cells, dCas13b‐M3 decreased cell proliferation and induced cell apoptosis. CRIPSR/dCas13b‐METTL3 is a newly developed method that targets methylation of specific mRNA in transcriptome to artificially manipulate cell homeostasis. Further, both in vitro and in vivo data suggested that ZNF677 was involved in m^6^A modification‐regulated growth of cancer cells. Given that ZNF677 possesses transcription factor activity, we thus consider that identification of its downstream targets is required to explore the mechanism underlying its regulation on RCC. In fact, a number of genes have been identified to be regulated by ZNF677 in NSCLC.^26^ Of them, CDKN3 have been demonstrated to promote the malignant progression of cervical cancer,^42^ regulate cisplatin resistance to colorectal cancer^43^ and promote cell proliferation and invasion in human ovarian cancer.^44^ In this study, clinical analysis confirmed the negative correlation between ZNF677 and CDKN3 in RCC tissues, and that high expression of CDKN3 reduced the survival rate of RCC patients. In addition, we also identified that CDKN3 was direct target of ZNF677 by a series of luciferase and ChIP‐qPCR assays. And CDKN3 knockdown inhibited RCC cells proliferation and rescued the antitumour phenotype impaired by ZNF677 deficiency. Therefore, in RCC cells, ZNF677 binds to the promoter of its target CDKN3 to regulate cellular activities, and is modified through an m^6^A‐dependent turnover mechanism by interacting with the m^6^A readers IGF2BP2 and YTHDF1.

## 4 CONCLUSION

Our findings indicate that ZNF677 is frequently downregulated by m^6^A methylation of CDS region in RCC, and demonstrate that methylation of CDS is critical for m^6^A‐mediated mRNA stability and translation. Further, we provided compelling in vitro and in vivo evidences demonstrating that m^6^A can regulate the tumour growth of RCC via regulation of ZNF677 expression. And we demonstrated that ZNF677 plays its tumour suppressor role in RCC through transcriptionally repressing its downstream target CDKN3. Importantly, our results revealed that m^6^A modification has vital role in regulating RCC progression and may be novel target for cancer therapy and diagnosis. The regulatory network involving the new complex METTL3/ZNF677/CDKN3 might provide new insight into the potential mechanism of the pathogenesis and development of RCC.

## 5 METHODS

### 5.1 Patients and tissue specimen collection

RCC samples and adjacent nonmalignant renal tissues with patients’ informed consent were obtained from the Urology Department of Peking University First Hospital (PKUFH), Beijing, China. This study followed the Helsinki declaration and was approved by the Institutional Ethical Review Board of PKUFH. The pathological diagnosis was made by professional urological pathologists. Samples were collected immediately in the operating room after surgical removal and were stored in liquid nitrogen after rapid freezing in liquid nitrogen for the following RNA isolation. We used these samples for a later mRNA and protein analysis.

### 5.2 RNA m^6^A and mRNA sequencing

MeRIP‐seq and RNA‐seq were performed by Cloudseq Biotech, Inc. (Shanghai, China), as described previously.^45^ Briefly, total RNAs were isolated from five pairs of tumours and adjacent tissues using trizol (Thermo Fisher Scientific). Then total RNA was broken into almost 100 nt fragmentation and were incubated with anti‐m^6^A antibody (Synaptic Systems, 202003, Goettingen, Germany) for 2 h at 4°C. Then the beads were prepared (Thermo Fisher Scientific) and incubated with the total RNA for 2 h at 4°C. Finally, the mixture was washed and purified with the m^6^A‐bound RNA with TE buffer. The samples after purification can be used to construct the library by NEBNext Ultra RNA Library Prep Kit (New England Biolabs, MA, USA) on Illumina HiSeq sequencer (Illumina, CA, USA). Raw data of RNA‐seq and m^6^A‐seq have been uploaded to NCBI database. These data can be found at https://www.ncbi.nlm.nih.gov/bioproject/PRJNA719065

### 5.3 Sequencing data analysis

After obtaining the sequencing data of control and IP samples, the read segment data should be preprocessed (such as filtering the read segment with poor sequencing quality), and then all the read segment sequence mapping of the two samples should be positioned on the reference genome, which is the basis of subsequent data processing and analysis. Then there were many read segments captured by methylation sites in the IP samples, which would be mapped to the reference genome to form a reading segment enrichment region or a ‘peak’ near the methylation sites. Therefore, the methylation enrichment point detection algorithm is called the peak calling algorithm. The m^6^A methylated peaks among the transcripts were identified by MACS,^46^ and metagene m^6^A distribution was researched by MetaPlotR.^47^ The DMGs were identified by diffReps.^48^ To explore the DMGs and DEGs from MeRIP‐seq and RNA‐seq, the Gene Ontology (GO) analysis and KEGG pathway enrichment analysis were performed.

### 5.4 RNA‐binding protein immunoprecipitation (RIP)

RIP assays were performed using a Magna RIPTM RNA‐Binding Protein Immunoprecipitation Kit (Millipore) according to the manufacturer's protocol. Briefly, the cells were collected and lysed in a complete radioimmunoprecipitation assay buffer containing a protease inhibitor cocktail and RNase inhibitor. Antibodies (5 μg) were pre‐bound to protein A/G magnetic beads in immunoprecipitation buffer (20 mM Tris‐HCl pH 7.5, 140 mM NaCl, 0.05% Triton X‐100) for 2 h and then incubated with 100 μl of cell lysate overnight at 4°C with rotation. RNA was eluted from the beads by incubation with 400 μl of elution buffer for 2 h, precipitated with ethanol and dissolved in RNase‐free water. The enrichment of certain fragments was determined by real‐time PCR.

### 5.5 RT‐qPCR, RIP‐qPCR and MeRIP‐qPCR

Total RNA of RCC tissues and cell lines were extracted using an RNA‐easy Isolation Reagent (Vazyme Biotech, Nanjing, China) according to the instructions, respectively, as previously described. The fragmented RNA was incubated with anti‐m^6^A antibody‐coupled beads. The m^6^A‐containing RNAs were then immunoprecipitated with IGF2BP2 or YTHDF1 and eluted from the beads. Both input control and m^6^A‐IP samples were subjected to RT‐qPCR with gene‐specific primers. cDNA was synthesised using HiScript III RT SuperMix for qPCR (Vazyme Biotech, Nanjing, China). qRT‐PCR was performed using spectrophotometry (ABI Prism 7500TM instrument, Applied Biosystems) with universal SYBR Green qPCR Master Mix (Vazyme Biotech, Nanjing, China). Glyceraldehyde 3‐phosphate dehydrogenase (GAPDH) was used as reference gene. Primers are listed in Table S1.

### 5.6 Chromatin immunoprecipitation assay

The ChIP assay was used to evaluate transcription factor ZNF677 binding to its target DNA using the Pierce Magnetic ChIP Kit (Pierce Biotechnology). The protocol was performed as described previously.^49^ Briefly, the cells were cross‐linked using 1% formaldehyde and then were lysed in SDS lysis buffer. The sonicated cell lysates were then mixed with chip dilution buffer and precleared with protein A‐agarose/salmon sperm DNA for 30 min. The recovered supernatant was incubated with either an anti‐ZNF677 antibody (Invitrogen, USA) or an isotype control IgG overnight at 4°C. Next, immunoprecipitated complexes were precipitated and washed. Cross‐linking of immunoprecipitates was reversed at 65°C for 4 h, which was followed by treatment with RNase A and 100 μg/ml proteinase K at 50°C for 3 h for DNA fragment recycling. Extracted DNA samples were finally dissolved in TE buffer and subjected to PCR analysis. The data were normalised by respective 5% input. Each experiment was performed in triplicate. Primers are listed in Table S1.

### 5.7 Protein isolation and Western blot

Total protein of cells was extracted by KeyGEN Bio TECH protein extraction kit (KGP1100) and separated on 10% SDS‐PAGE and transferred onto nitrocellulose membrane. After blocking, blots were immunostained with primary and secondary antibodies, respectively, as previously described. The antibodies were as follows: ZNF677 (1:1000; Invitrogen, USA), METTL3 (1:1000; Invitrogen, USA), YTHDF1 (1:1000; Proteintech, USA), IGF2BP2 (1:1000; Invitrogen, USA), CDKN3 (1:1000; Abcam, USA) and GAPDH (1:10 000; Proteintech, USA). Immunohistochemistry staining was performed using a primary antibody of ZNF677 at a 1:300 dilution following a protocol described previously. All photographs were taken randomly and measured using Image Pro Plus (Media Cybernetics, Rockville, MD, USA).

### 5.8 Expression plasmids, short interfering RNAs and lentivirus transfection

The CRISPR dCas13b plasmids and Cas13b‐gRNA plasmids were purchased from Addgene. All designed gRNA and dCas13b‐METTL3 vector were constructed by Synbio Technologies Company (Suzhou, China). The CDS of METTL3, IGF2BP2 and YTHDF1 were cloned into pcDNA3.1 to generate overexpression plasmid. pcDNA3.1 was used as the vector control for analysis. The sequences are presented in Table S2. For ZNF677 and CDKN3 knockdown, synthesised duplex RNAi oligos and shRNAs targeting human mRNA sequences from Sigma were used. SiRNA and shRNA sequences are shown in Table S3.

### 5.9 Cell culture and plasmid transfection

RCC cell lines (786‐O, 769‐P, CAKI1, CAKI2 and OSRC) were used in this study. HK‐2 human kidney proximal tubular epithelial cells were used as normal controls. These cell lines were purchased from the American Type Culture Collection (ATCC, Manassas, VA, USA) and National Infrastructure of Cell Line Resource, China. Cell lines were routinely cultured in RPMI 1640 or DMEM, which was supplemented with 10% fetal bovine serum (Invitrogen, Carlsbad, CA, USA) and incubated in a 5% CO_2_ environment at 37°C. All plasmids and siRNAs were transfected with lipo3000 (Invitrogen) following manufacturer's protocol and 1 μg of plasmids was used in the experiments. The working concentration of siRNA was 50 nM.

### 5.10 Luciferase reporter assay

To evaluate the effect of CDS on ZNF677 expression, the wild‐type or mutant‐1/‐2/‐3/‐4 of CDS of ZNF677 was inserted behind the F‐luc coding region. Both the pmirGLO‐ZNF677‐CDS‐WT and pmirGLO‐ZNF677‐CDS‐Mut‐1/‐2/‐3/‐4 were transfected into wild‐type or Mettl3 overexpression cells for 24 h, the firefly luciferase (F‐luc) and Renilla luciferase (R‐luc) were assayed by Dual‐Glo Luciferase Assay system (Promega). R‐luc was used to normalise F‐luc activity. Promoter activity of CDKN3 in cells was measured by luciferase assay according to our previously described protocol.^50^ Briefly, cells were transfected with pGL3‐CDKN3‐WT‐Luc, pGL3‐CDKN3‐Mut1‐Luc or pGL3‐CDKN3‐Mut2‐Luc. After 24‐h incubation, luciferase activity was measured using the Dual Luciferase Reporter Assay kit (Promega) according to the manufacturer's instructions. R‐luc was used to normalise F‐luc activity to evaluate the reporter transcription. Experiments were performed three times with similar results.

### 5.11 mRNA stability

Stability of RNA in cells transfected with different plasmids was achieved by incubating cells with actinomycin D (Act‐D, Catalog #A9415, Sigma, USA) at 5 μg/ml. Cells were then collected at the indicated times and RNA was isolated for real‐time PCR. Half‐life (*t*_1/2_) of ZNF677 mRNA was calculated using ln2/slope and GAPDH was used for normalisation.

### 5.12 Protein stability

Protein stability of targets in cells transfected with different plasmids was achieved by incubation of CHX (final concentration 100 μg/ml) during indicated times. The expression of ZNF677 was measured through Western blot analysis.

### 5.13 Cell proliferation and apoptosis assays

Cell proliferation was detected by CCK8 assay (Transgen, China). For each well, 3 × 10^3^ cells were seeded into 96‐well plates. The cells were cultured for 24, 48, 72 and 96 h, and incubated with CCK8 at 37°C for 3 h. Then the absorbance at 450 nm was measured with a microplate reader. Cells transfected with vectors were inoculated on a 12‐well plate (2 × 10^5^ cells/well) with 70%–80% confluency. After 48 h, the cell was detected by the caspase‐3/ELISA (enzyme‐linked immunosorbent assay) (Hcusabio, China). The caspase‐3 enzyme is a marker for inflammation and apoptosis signaling, as it can regulate the destruction of DNA or cytoskeletal proteins. Each test was performed at least three times.

### 5.14 Xenograft models

BALB/c nude female mice (4–5 weeks old) were randomly divided into four groups (six per group) and housed under standard conditions. Stably transfected OSRC cells were subcutaneously injected into the right flanks of the mice using 5 × 10^6^ cells per mouse. Tumour volumes were measured every week. Four weeks after injection, the mice were humanely euthanased. Subcutaneous tumour tissues were isolated, and the weights of the dissected tumours were measured. All animal experiments were approved by the animal management committee of Peking University Shenzhen Hospital, and all experimental procedures and animal care were in accordance with the institutional ethics guidelines for animal experiments.

### 5.15 Database (DB) analysis

Kaplan–Meier plotter (http://kmplot.com/analysis/)^51^ was used to assess the prognostic value of ZNF677 and CDKN3 expression in patients with RCC. Expression levels of ZNF677, IGF2BP2 and CDKN3 in cancer tissues and normal tissues of RCC were obtained from TCGA database.^52^ The correlation between ZNF677 and CDKN3 was evaluated by use of LinkedOmics (http://www.linkedomics.org), which is a publicly available portal that includes multi‐omics data from all 32 cancer types from TCGA.

### 5.16 Statistical analyses

Data were reported as mean ± SD from at least three independent experiments. For statistical analysis, two‐tailed unpaired Student's *t*‐tests between two groups and by one‐way or two‐way ANOVA followed by Bonferroni test for multiple comparisons were performed. All statistical tests were two‐sided. Data analysis was carried out using SPSS 16.0 for Windows. A *p*‐value of <.05 was considered to be statistically significant; *p < .05, **p < .01; NS, not significant.

## CONFLICT OF INTEREST

The authors declare no conflict of interests regarding the publication of this paper.

## Supporting information

Supporting Information

Supporting Information

Supporting Information

Supporting Information

Supporting Information

Supporting Information

Supporting Information

Supporting Information

Supporting Information

Supporting Information
