# Supplementary figures and images for: ZNF677 suppresses renal cell carcinoma progression through N6‐methyladenosine and transcriptional repression of CDKN3
Source: Clin Transl Med. 2022 Jun 9;12(6):e906. doi: 10.1002/ctm2.906 (PMC9178504; doi:10.1002/ctm2.906)

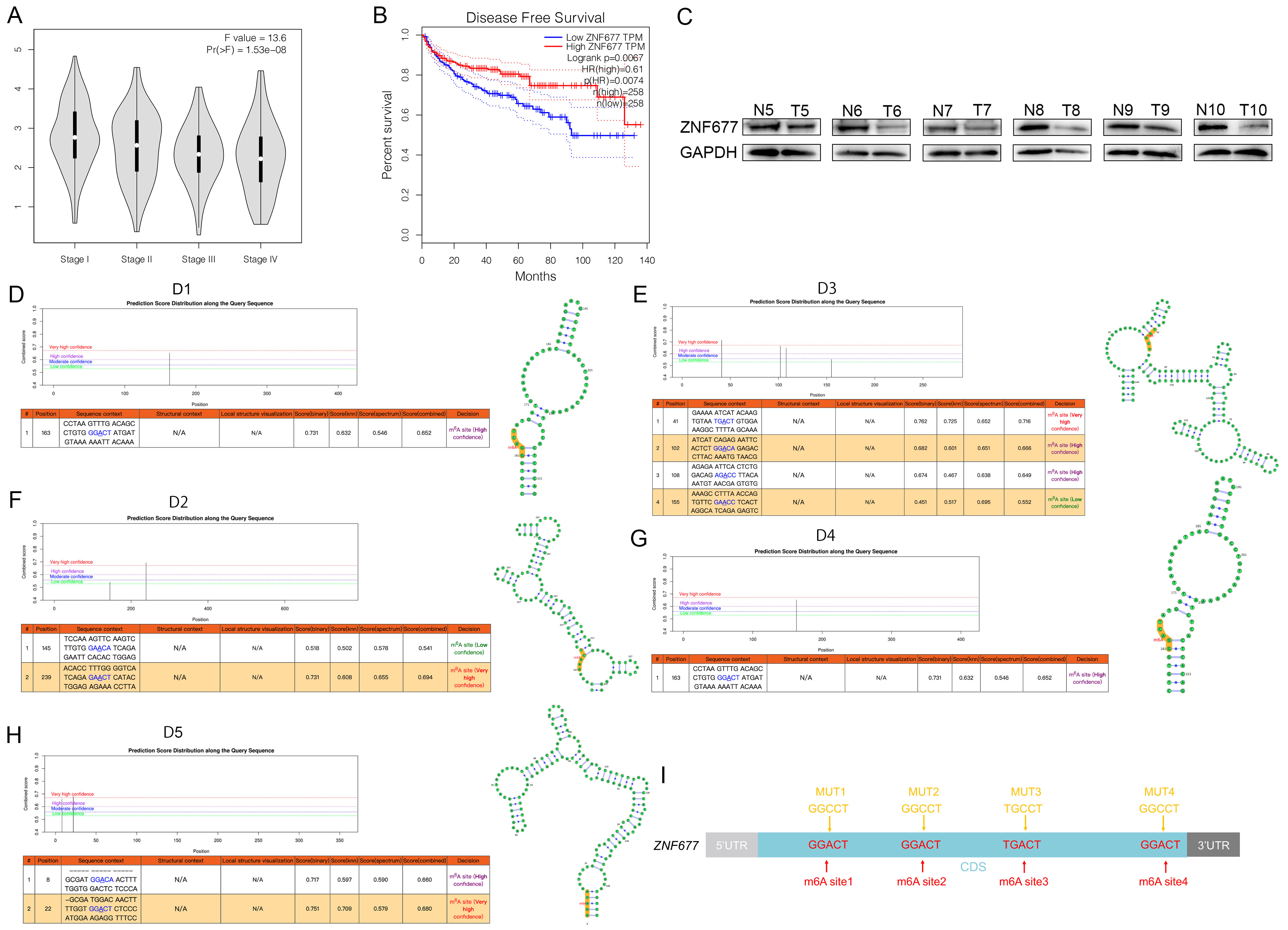

Supplement: Supplementary file 1 — Supporting Information [file CTM2-12-e906-s001.jpg]

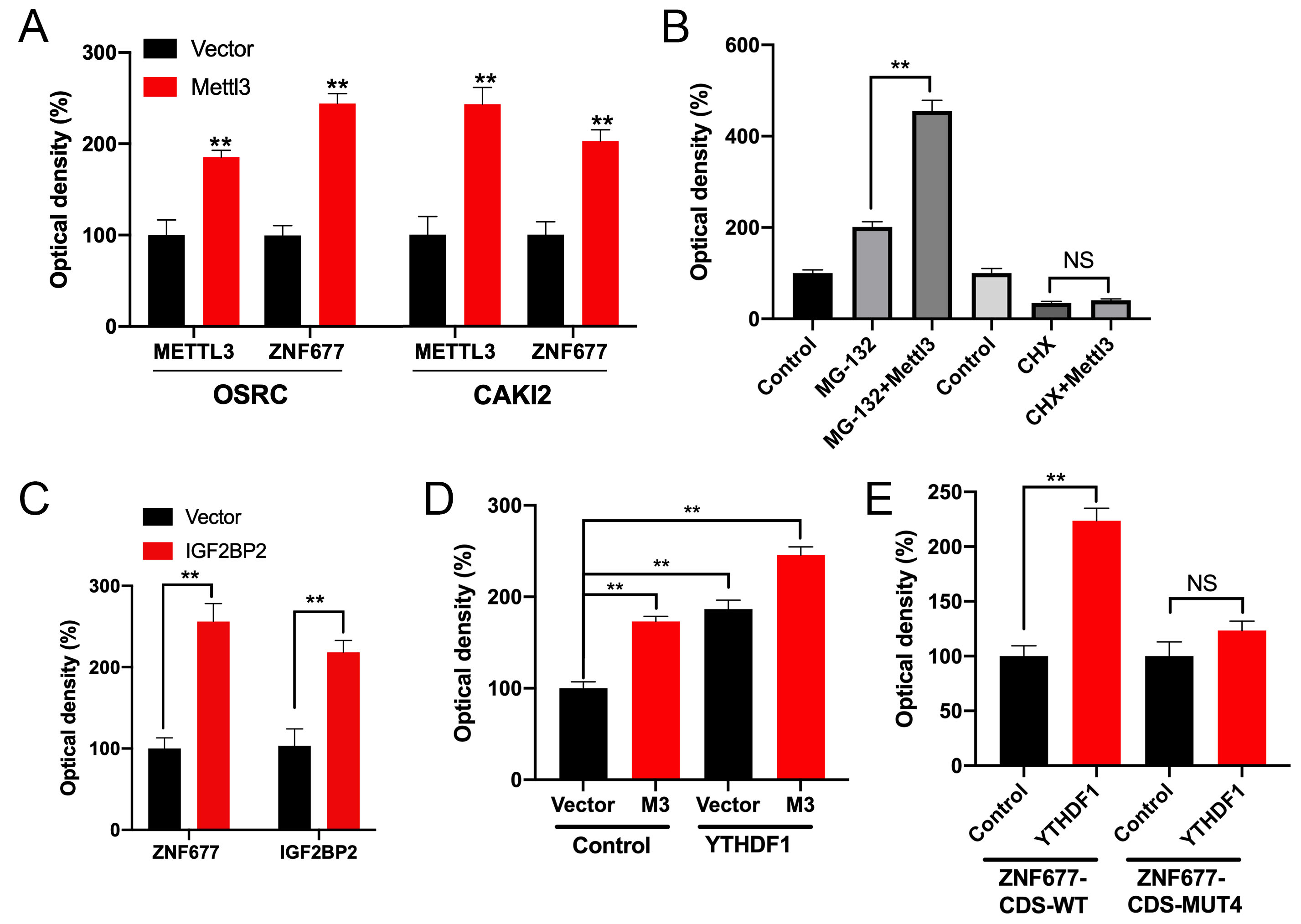

Supplement: Supplementary file 2 — Supporting Information [file CTM2-12-e906-s003.jpg]

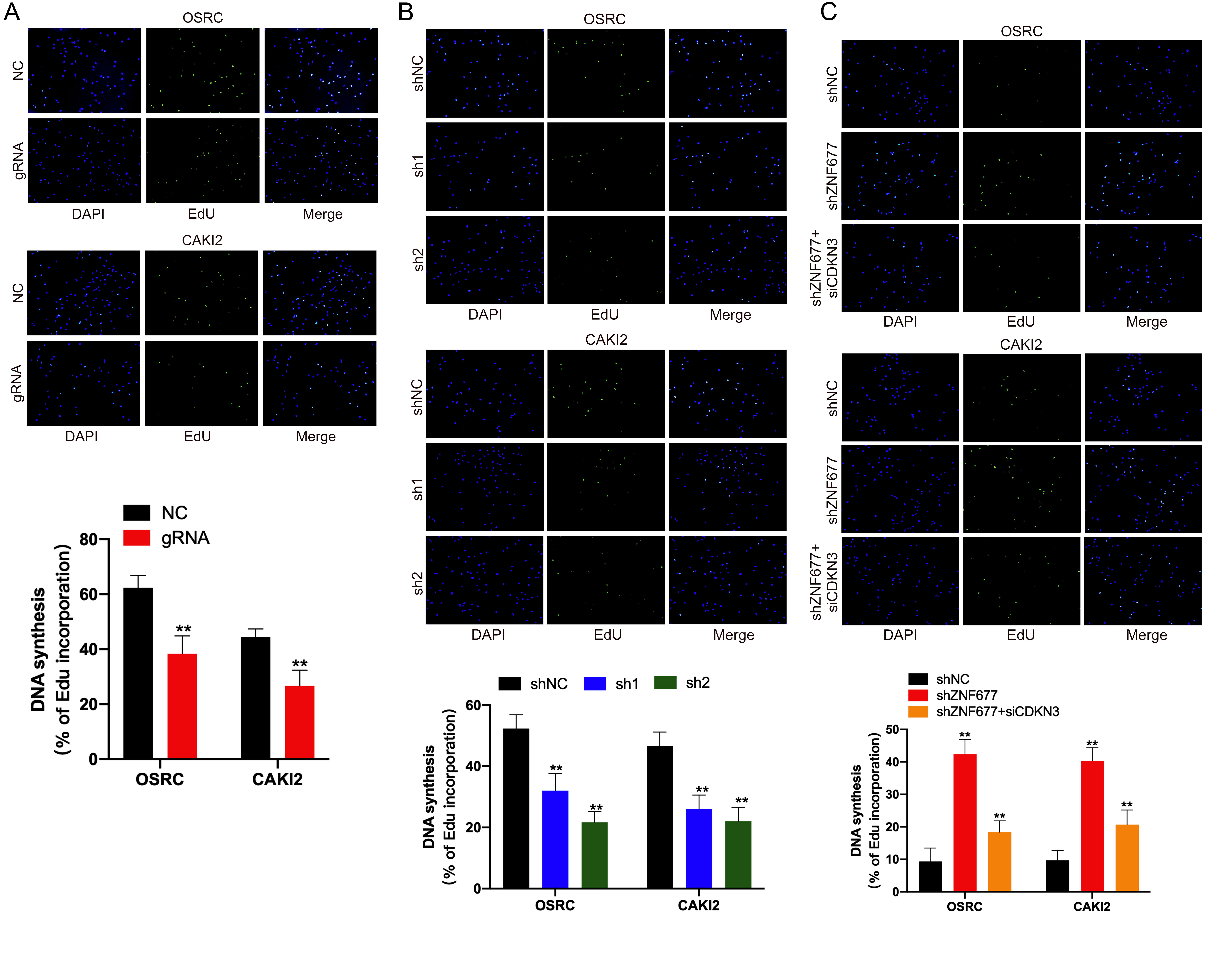

Supplement: Supplementary file 3 — Supporting Information [file CTM2-12-e906-s009.jpg]

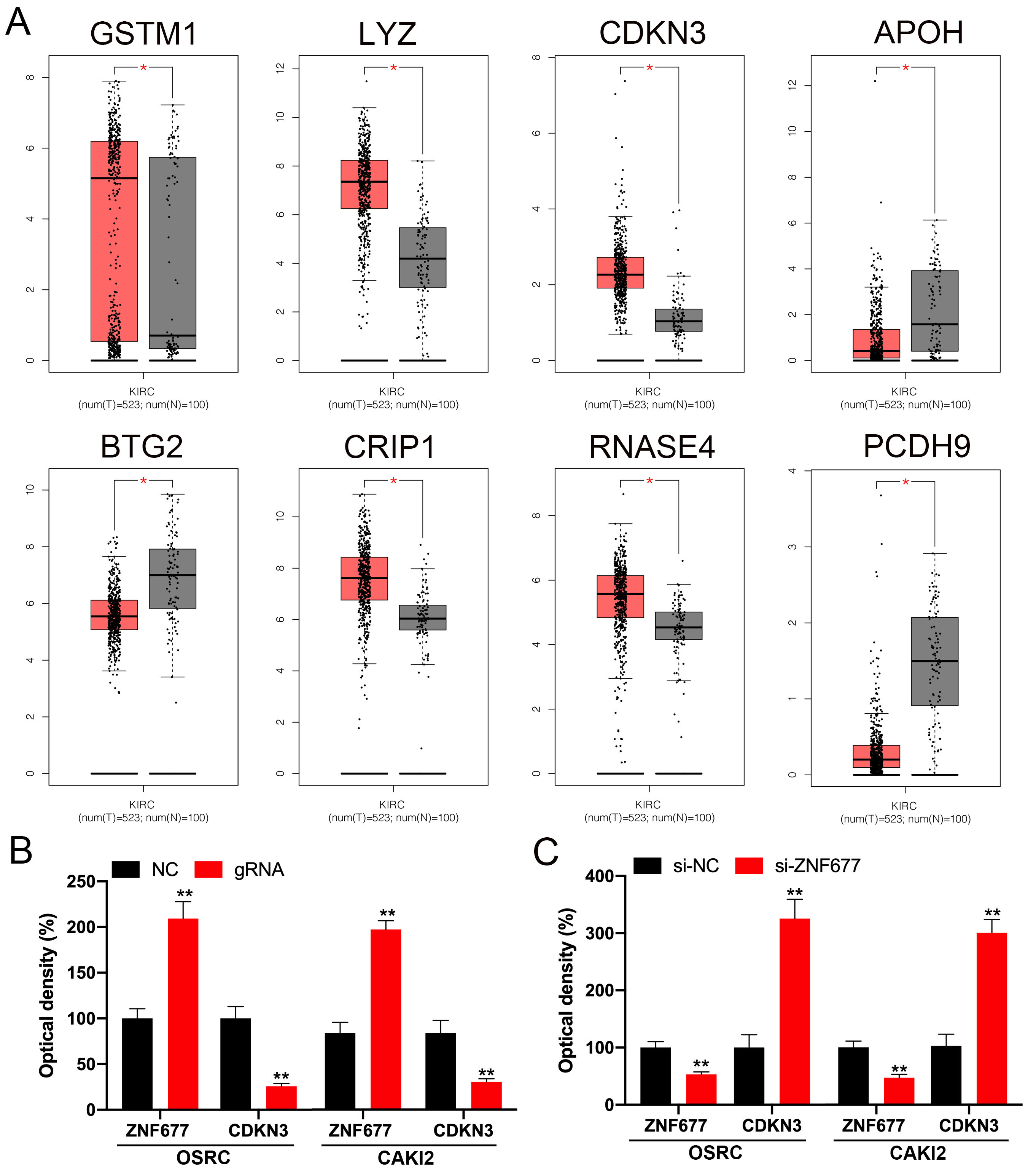

Supplement: Supplementary file 4 — Supporting Information [file CTM2-12-e906-s008.jpg]

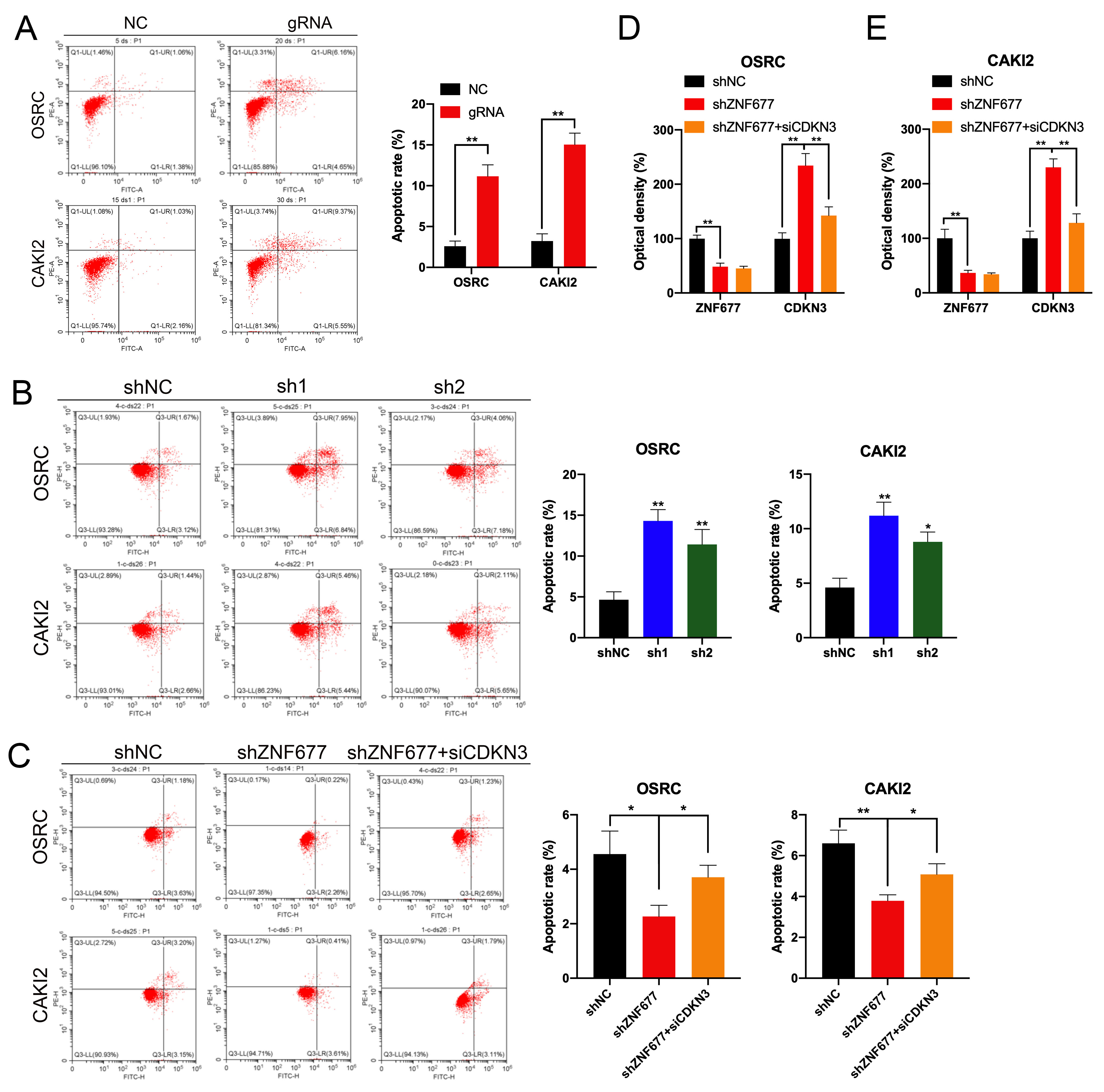

Supplement: Supplementary file 5 — Supporting Information [file CTM2-12-e906-s006.jpg]

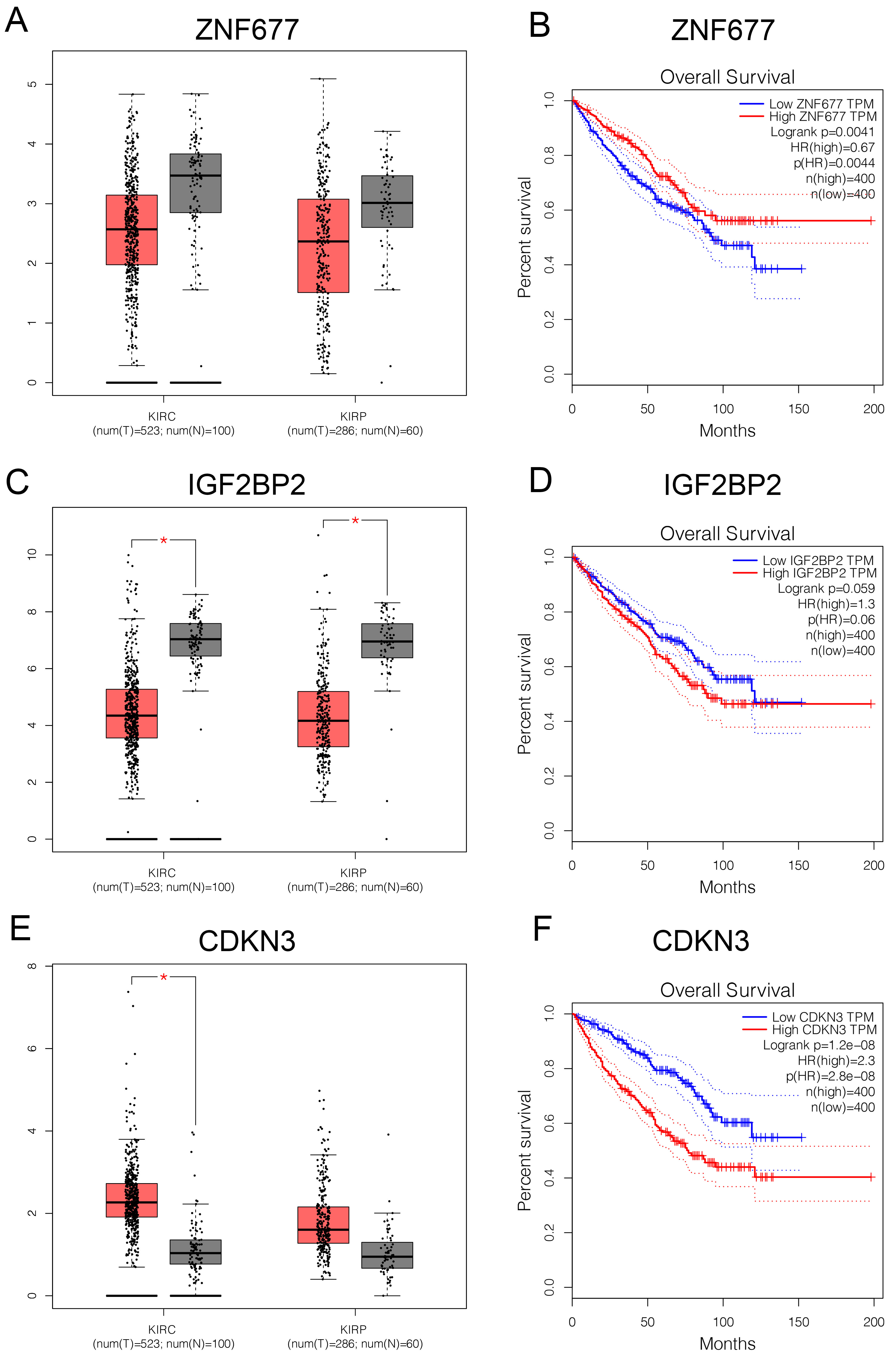

Supplement: Supplementary file 6 — Supporting Information [file CTM2-12-e906-s005.jpg]
